# An Epidemiological Study on Legionnaires’ Disease in Gyeonggi Province, Korea: Occurrence, Infection Routes, and Risk Factors for Mortality (2016–2022)

**DOI:** 10.3390/medicina60020227

**Published:** 2024-01-28

**Authors:** Hae-Deun Noh, Jeonghyeon Oh, Kun-Hee Park, Boyoung Park

**Affiliations:** 1 Gyeonggi Infectious Disease Control Center, Health Bureau, Gyeonggi Provincial Government, Suwon 16508, Republic of Korea; shgoems@gmail.com (H.-D.N.); jhyeon.paz@gmail.com (J.O.); kpark.ghealth@gmail.com (K.-H.P.); 2 Department of Preventive Medicine, Hanyang University College of Medicine, Seoul 04763, Republic of Korea

**Keywords:** Legionella, mortality, community-acquired infections, nosocomial infections

## Abstract

*Background and Objectives*: Legionnaires’ disease (LD) is an acute respiratory disease with increasing annual numbers of reported domestic and global cases. This study aimed to establish foundational data for the prevention and control of LD by investigating the occurrence and infection routes of reported and suspected cases of LD in Gyeonggi Province, Korea, from January 2016 to December 2022, and by and analyzing the risk factors for death. *Materials and Methods*: A sex-and-age standardization was performed on LD patients and suspected cases reported in Gyeonggi Province. The monthly average number of confirmed cases was visualized using graphs, and a survival analysis was performed using Kaplan–Meier survival curves. The mortality risk ratio was estimated using the Cox proportional hazards model. *Results*: The incidence of LD in Gyeonggi Province mirrored the national trend, peaking in July with the highest number of confirmed and suspected cases. While there was no significant difference in survival rates by age, the survival rate was higher for suspected cases when analyzed separately. Comparing the death ratio by infection route, nosocomial infections showed the highest death ratio, and intensive care unit (ICU) admission and the presence of coinfections were significantly correlated with mortality. Factors such as nosocomial infection, admission within 1 to 3 days following diagnosis, and the development of complications were factors contributing to a higher risk of death. *Conclusions*: The general characteristics of patients with LD were similar to those suggested by previous studies. The proportion of community-acquired infections was lower than in previous studies, but the length of hospital stay was similar for survivors and the deceased, and the mortality rate within 30 days after diagnosis was higher for nosocomial infections. In conclusion, nosocomial infection, a period of up to 3 days from admission to diagnosis, and complications were significantly related to the mortality rate of LD.

## 1. Introduction

Legionnaires’ disease (LD), an acute respiratory disease caused by *Legionella* spp., was first identified in 1976 during an epidemiological investigation into a mass pneumonia outbreak among a US cohort attending an annual meeting in Philadelphia, USA [1].

In the United States, the number of LD cases surged by 192%, from 1110 in 2000 to 3522 in 2009. The actual incidence is presumed to be higher due to the reliance on a passive surveillance system based on reports from healthcare workers and laboratory results [2]. A study exploring LD-related mortality from 2000 to 2010 revealed an increase in deaths from 0.038 to 0.040 per 100,000 people, with a twofold higher rate in men. Underlying conditions such as leukemia and rheumatoid arthritis were found to be associated with LD-related deaths [3]. In Europe, the reported incidence among European Union/European Economic Area member states has been increasing in number annually, from 1.4 per 100,000 in 2016 to 2.2 per 100,000 in 2019 [4]. Among the total reported cases in Europe (*n* = 23,164) between 2011 and 2015, 9.3% resulted in death, with no significant difference between sexes [5].

In Korea, the annual number of LD cases remained below 10 from 2000 to 2005 and between 20 and 30 cases from 2006 to 2014. A noticeable rise in the incidence rate began around 2015, escalating from 0.25 per 100,000 in 2016 to 0.74 per 100,000 in 2021 [6].

Previous studies have commonly identified Legionella bacteria in patients hospitalized with pneumonia in community settings [7,8,9]. Considering the essential role of water in residential settings for activities like showering and bathing, the potential for community-acquired Legionella infections is always present.

Despite the upward trend in Legionella infections both domestically and globally, there are no adequate studies on the mortality risk factors associated with LD in Korea. This study aimed to generate critical epidemiological data to guide the development of policies for LD prevention and control. It focuses on examining the recent incidence and infection routes of reported and suspected LD cases in Gyeonggi Province from January 2016 to December 2022 and on analyzing the risk factors of mortality due to LD.

## 2. Materials and Methods

### 2.1. Case Definition

The study population included 543 individuals reported as confirmed or suspected cases of Legionellosis in Gyeonggi Province from January 2016 to December 2022 via the Integrated Disease Health Management System (https://is.kdca.go.kr; accessed on 01/07/2023) operated by the Korea Disease Control and Prevention Agency (KDCA). According to the 2023 Legionellosis management guidelines released by the KDCA [6], “a confirmed case of Legionella infection” is defined as “a case in which the patient has clinical symptoms characteristic of legionellosis and meets the laboratory testing criteria for a confirmed diagnosis”. “A suspected case of Legionella infection” is defined as “a case in which the patient is suspected to have legionellosis based on clinical symptoms and epidemiological relevance, and presumed to be infected with Legionella based on the laboratory testing criteria for a suspected diagnosis”. A confirmed case is established if one or more of the following three testing criteria are met: (1) isolation of Legionella bacteria from specimens such as bronchoalveolar lavage, bronchial aspirate, sputum, lung tissue, pleural fluid, and blood; (2) detection of specific antigens in urine samples; (3) a ≥ fourfold increase in antibodies in convalescent serum compared to the acute phase. A suspected case is established if one or more of the following three testing criteria are met: (1) detection of specific antigens in specimens through direct fluorescence antibody tests; (2) a single antibody titer of 1:128 or higher in blood samples through indirect fluorescence antibody tests; (3) detection of specific genes in the specimen. Those who did not meet the criteria for testing confirmed and suspected cases were not included. The case definitions for Legionella patients and suspected cases are summarized in Table 1.

LD-related deaths are defined as those occurring within 30 days following the diagnosis of the infection.

### 2.2. Data Source

For this study, the following variables were obtained from the epidemiological investigation reports written by the epidemiologists in Gyeonggi Province:

1. General characteristics (age, gender)
2. Clinical characteristics

- *Clinical symptoms*: For confirmed and suspected cases, the medical records of individuals with a history of visits to a healthcare facility were reviewed. Those with one or more of the following clinical symptoms were classified as “Yes”: fever, chills, cough, general fatigue, headache, myalgia, arthralgia, chest pain, loss of appetite, hemoptysis, dyspnea, nausea, vomiting, consciousness disorder, diarrhea, and sore throat. Asymptomatic cases were classified as “No”. For individuals with no medical records, classification was made through interviews.
- *Healthcare facility utilization history*: For individuals who could be interviewed, a direct telephone interview was conducted. If communication with the patient or suspected case was impeded, the caregiver or public health center was consulted. Upon confirmation of a healthcare facility utilization history, the facility was visited for medical record review to verify the visit history, diagnosis upon admission, length of hospital stay, and reason for admission through medical records.
- *Antibiotic use history*: For patients and suspected cases with a healthcare facility visit history, medical records were reviewed to check the antibiotic use history. If either macrolide, quinolones, tetracycline, rifampin, or other antibiotics were used, the case was classified as “Yes”. If no antibiotics were used, it was classified as “No”.
- *Complications*: For patients and suspected cases with a healthcare facility visit history, medical records were reviewed to check the presence of complications. Cases with respiratory failure, renal failure, multiple organ failure, neurological deficit, lung abscess, or empyema were classified as “Yes”. Cases showing none of these symptoms were classified as “No”. Cases of missing data or uncertainties regarding the occurrence of complications at the time of the investigation were classified as “Undetermined”.
- *Coinfection*: For patients and suspected cases with a healthcare facility visit history, medical records were reviewed to check the presence of pathogens other than *Legionella* spp., as confirmed through the laboratory testing of clinical specimens. Depending on the presence or absence of such pathogens, cases were classified as “Yes” or “No”.
- *Time lag between symptom onset and diagnosis:* The duration from the onset of symptoms to the day of diagnosis was calculated for all patients and suspected cases.
- *Hospital admission-to-diagnosis time lag*: The duration from the date of hospital admission to the date of diagnosis was calculated for hospitalized patients and suspected cases.
- *Mortality*: For patients and suspected cases with a healthcare facility visit history, medical records were reviewed to check mortality. Depending on whether death occurred within 30 days of admission or not, cases were classified as “Yes” or “No”.

3. Risk factors

Interviews were conducted directly over the phone with individuals who were available and able to participate. In cases where individuals could not be interviewed due to unconsciousness or other reasons, information was obtained from medical records based on data collected at the time of admission to healthcare facilities.

- *Underlying diseases*: Cases were classified as “Yes” if they had one or more of the following chronic diseases: chronic pulmonary disease (chronic obstructive pulmonary disease, asthma, tuberculosis, others), immunological disorders (autoimmune diseases, acquired immunodeficiency syndrome, others), blood disorders (aplastic anemia, others), or other chronic diseases (cancer, chronic renal failure, diabetes, others). Cases without any underlying diseases were classified as “No”.
- *Alcohol consumption*: Current drinkers were classified as “Yes” and former or never-drinkers as “No”. Among the current drinkers, the average amount of soju consumed daily and the frequency of drinking per week were examined.
- *Smoking status*: Current smokers were classified as “Yes” and former or never-smokers as “No”. Among the current smokers, the average number of cigarettes smoked per day and the duration of smoking (in years) were examined.

4. Exposure factors (infection routes)

Exposure factors were classified based on the definitions of infection routes as specified in the 2023 Legionellosis management guidelines [6]:

- *Community-acquired infection*: This category encompasses infections that are not hospital-acquired or travel-related. These infections occur in settings where exposure to water systems is suspected, typically within 2 weeks before the onset of symptoms. Examples include workplaces, large buildings, department stores, swimming pools, and saunas. Additionally, domestic infections fall under this category, arising from continuous residence and use of home water systems within 2 weeks prior to symptom onset.
- *Nosocomial infection:* Classification is based on the length of hospital stay prior to the onset of Legionellosis, along with environmental investigation findings, with a focus on hospitalization within 10 days before symptom onset.
- *Other*: This category encompasses infections that are travel-related, occurring after an overnight stay in Korea or abroad within 2 weeks prior to symptom onset, as well as cases where the route of infection is unknown or falls outside the definitions of nosocomial or community-acquired infections.

### 2.3. Statistical Analysis

Indirect standardization of LD incidence by age groups in Gyeonggi Province and across the country was performed using SAS statistical software (version 9.4, SAS Institute, Cary, NC, USA). A comparison was performed between LD patients and suspected cases reported in Gyeonggi Province from 2016 to 2022 and those among the nationwide registered population after sex and age standardization by categorizing the subjects by sex and age segmented at 5-year intervals. The monthly average number of confirmed cases was visualized using Microsoft Excel 2019 MSO version 16.0.10827.20150 (Microsoft Corporation, Redmond, WA, USA), 64-bit. Statistical analysis was conducted using R statistics software (version 1.4.1106, R Foundation for Statistical Computing, Vienna, Austria), and the chi-square test was used to determine the differences in frequency as descriptive statistics.

For survival analysis, the follow-up period was defined as the period from the date of diagnosis to the date of death for deceased patients and from the date of diagnosis to the date of discharge for survivors. Kaplan–Meier analysis was conducted for survival analysis. In the Kaplan–Meier survival curves, the *x*-axis represents the days from the diagnosis, applying a follow-up period of up to 30 days, and the *y*-axis represents the survival probability. For one patient whose date of death was uncertain, an average follow-up period of 6 days was applied, and excluding this patient did not change the results. Factors influencing mortality were estimated using the Cox proportional hazards model. A univariate analysis was conducted, and all variables included in the univariate analysis were also included in the multivariate Cox regression analysis. For all analysis results, a *p*-value < 0.05 was considered statistically significant.

### 2.4. Ethics Statement

In compliance with ethical principles, this study was conducted after obtaining approval from the Ministry of Health and Welfare’s designated Institutional Review Board (Approval Number: P01-202305-01-027).

## 3. Results

### 3.1. Incidence of LD

The standardized incidence ratio was 1.07 (95% confidence interval (CI) = 0.98–1.16, *p* value = 0.12), indicating that the incidence of LD in Gyeonggi Province was similar to the national incidence.

### 3.2. Monthly Incidence

**Figure 1:**
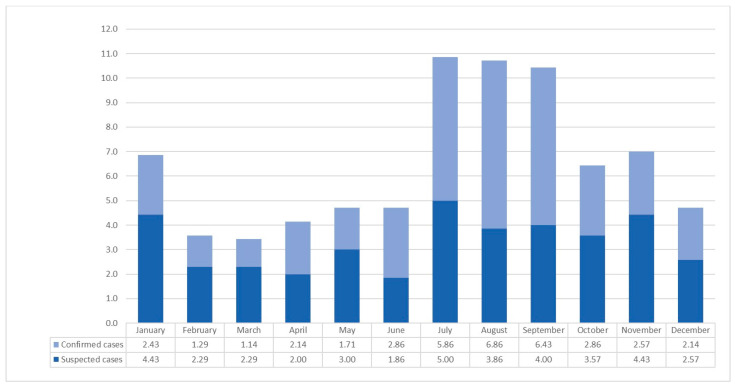
Average monthly number of confirmed and suspected Legionnaires’ cases in Gyeonggi-do from 2016 to 2022.

Over the 7-year period from 2016 to 2022, the average monthly number of LD patients and suspected cases in Gyeonggi Province varied significantly: 6.86 in January, 3.58 in February, 3.43 in March, 4.14 in April, 4.71 in May, 4.72 in June, 10.86 in July, 10.72 in August, 10.43 in September, 6.43 in October, 7.00 in November, and 4.71 in December. March and July had the lowest and highest incidence rates, respectively. It is worth noting that the highest number of patients with LD (6.86) was recorded in August, while the highest number of suspected cases (5.00) was observed in July (Figure 1).

### 3.3. Basic Characteristics

Table 2 outlines the basic characteristics of survival and mortality regarding LD patients and suspected cases from 2016 to 2022 in Gyeonggi Province. Out of 543 patients and suspected cases, 488 survived and 55 died. There was no significant age-dependent difference among the total number of patients. However, when broken down into patients and suspected cases, the frequency of deaths was higher among patients aged 60–69 compared to those under 60, and among suspected cases, the frequency of deaths was significantly higher among those aged 70–79 (patients *p* = 0.032, suspected cases *p* = 0.003). When broken down by infection route, community-acquired infections had 275 cases, higher than nosocomial infections and others. However, when examining the proportion of deaths in each category, nosocomial infections had the highest mortality rate (20.27%, *p* = 0.001). Of the 497 patients investigated for intensive care unit (ICU) admission, 160 received ICU treatment, and 34 (21.25%) died, which was a higher mortality rate compared to those who did not receive ICU treatment (patients *p* < 0.001, suspected cases *p* < 0.001). Among the 153 patients with coinfections out of 503 investigated cases, 23 (15.03%) died, a higher mortality rate than those without coinfections (*p* = 0.022). Regarding alcohol consumption, the mortality rate was higher among nondrinkers (11.97%) compared to drinkers (3.42%, *p* = 0.006).

### 3.4. Survival Curve

**Figure 2:**
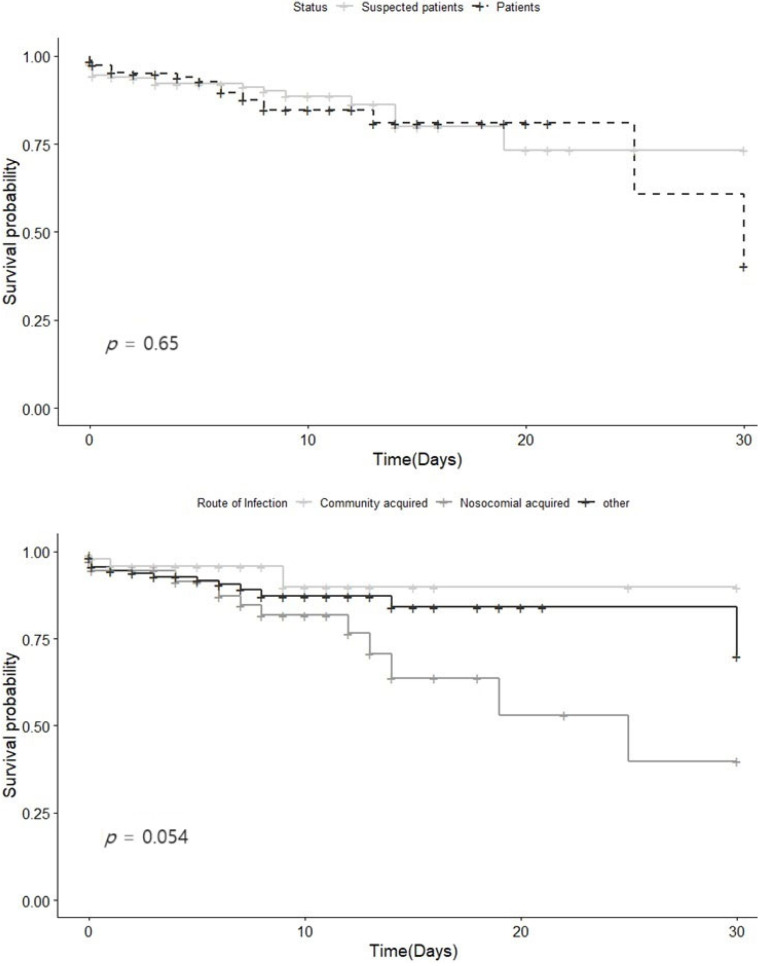
Survival curve for the confirmed and suspected Legionnaires’ cases in Gyeonggi-do from 2016 to 2022. (**a**) Stratified confirmed and suspected Legionnaires’ cases (**b**) by route of infection.

The 30-day survival rate from the date of diagnosis was 89.55% for patients and 90.18% for suspected cases. While the survival rate was slightly higher among suspected cases, the difference was not statistically significant. When examined by infection route, the 30-day survival rates were 93.45% for community-acquired infections, 88.66% for other infections, and 79.73% for nosocomial infections. While there was no significant difference among these three infection routes (*p* = 0.054), nosocomial infections showed a lower survival rate than the other categories (Figure 2).

### 3.5. Factors Associated with Survival

Univariate and multivariate analyses were conducted using the Cox proportional hazards model to identify factors associated with mortality (Table 3).

In the univariate analysis, nosocomial infections were associated with a 3.10-fold increased risk of death (95% CI = 1.12–8.56, *p* < 0.001) compared to community-acquired infections. Other infections were also associated with a higher risk of death, with a 1.81-fold increase compared to community-acquired infections (95% CI = 0.71–4.62, *p* = 0.029). Additionally, patients diagnosed within 1 to 3 days of admission exhibited an 11.03-fold increased risk of death compared to those diagnosed later (95% CI = 4.02–30.21, *p* < 0.005). The risk of death was 4.40 times higher for patients who developed complications compared to those who did not (95% CI = 2.19–8.86, *p* < 0.005). Furthermore, cases with missing or undetermined complications at the time of the epidemiological investigation had a 5.14-fold increased risk of death compared to those with clearly defined complications (95% CI = 1.60–16.54, *p* = 0.006). Interestingly, current drinkers had a 0.27-fold reduced risk of death compared to nondrinkers (95% CI = 0.10–0.74, *p* = 0.011). Additionally, antibiotic treatment for more than 3 days was associated with a 0.34-fold reduced risk of death (95% CI = 0.19–0.61, *p* < 0.001). Sex, age, smoking status, underlying diseases, and coinfections did not show a statistically significant association with mortality.

In the multivariate analysis, the factors affecting mortality were identified as (i) nosocomial infections, with a 5.23-fold increased risk of death (95% CI = 1.72–15.94, *p* = 0.004) compared to community-acquired infections; (ii) patients diagnosed within 1 to 3 days of admission, with a 4.39-fold increased risk of death (95% CI = 1.29–14.90, *p* = 0.02); (iii) patients who developed complications, with a 5.46 times higher risk of death than those who did not (95% CI = 2.64–11.28, *p* < 0.005); and (iv) cases with missing or undetermined complications at the time of the epidemiological investigation, with a 4.86-fold increased risk of death compared to those with clearly defined complications (95% CI = 1.42–16.67, *p* = 0.01). Factors associated with lower mortality rates were (i) alcohol consumption, with drinkers having 0.17 times lower mortality rate than nondrinkers (95% CI = 0.06–0.57, *p* = 0.003); and (ii) use of antibiotics, with those administered antibiotics for ≥ 3 days having 0.17 times lower mortality rates than those administered antibiotics for <3 days (95% CI = 0.08–0.39, *p* < 0.005). Sex, age, smoking status, and coinfections were found to have no statistically significant impact on mortality.

## 4. Discussion

From 2016 to 2022, the incidence of Legionella infections in Gyeonggi Province was similar to the national incidence during the same period, with the highest incidence recorded in July and August. The average age of survivors was 67.4 years, while the average age of those who died was 71.6 years. Although 50.6% of all cases occurred in the community, the proportion of deaths was about three times higher in nosocomial infections (20.27%) than in community-acquired infections (6.55%). In the Kaplan–Meier survival analysis, the survival rate of suspected cases was slightly higher than that of patients, though it did not reach statistical significance. By infection route, the survival rate of nosocomial infections was lower than in other categories but without significant difference. Risk factors for death included nosocomial infections, nondrinkers, a time lag of 1 to 3 days from admission to diagnosis, and complications. In contrast, the use of antibiotics for more than 3 days was associated with a lower risk of death.

Legionella infection showed a seasonal pattern, peaking from late summer to autumn, which aligns with previous studies [10,11,12,13,14,15,16,17]. In the gender distribution of global cases, males mostly outnumbered females [2,3,5,7,8,9,10,11,12,17,18,19,20,21,22], and the most frequent age range of patients was 50–70 years, similar to this study [5,9,10,11,12,14,18,23,24]. In this study, community-acquired infections accounted for 50.6% of all cases, lower than the values estimated in previous studies [5,10,21,24]. The results regarding the length of hospital stay for survivors and deceased patients were similar to those of a previous study [12], where the median length of hospital stay was 8 days for all patients, 20 days for survivors, and 8 days for deceased patients. Additionally, in terms of the 30-day mortality rate, the risk of death from nosocomial infections was more than twice as high as that from community-acquired infections, which aligns with the findings of previous studies [10,22]. The risk factors for developing LD and mortality identified in previous studies [10,11,13,16,18,19,21,25], including chronic bronchitis, diabetes, cancer, kidney disease, blood disorders, and neurological diseases, are consistent with the findings of this study. Regarding clinical symptoms, one study found that the main clinical symptoms in deceased patients were disturbance of consciousness, respiratory distress, and decreased blood pressure, while the main symptoms in survivors were fever, chills, abdominal pain, and headache [19]. A study mentioned fever, respiratory symptoms, and gastrointestinal symptoms [21], consistent with the findings of this study. In this study, the 30-day mortality rate for patients with Legionella pneumonia admitted to the ICU was 21.25%, lower than the 26.1% mortality rate reported in a previous study [12]. A study estimated the mortality rate for community-acquired infections at 10.2% and for nosocomial infections at 35.3% [10], while another study found no significant difference between the two infection routes, with a mortality rate of 30% for community-acquired infections and 27% for nosocomial infections [26]. The findings of this study also support the findings of previous studies that the risk factors for death among LD patients are complications, a shorter duration of antibiotic treatment for the deceased (duration of antibiotic treatment was, on average, 4 days for survivors and 3 days for the deceased) [5], age, ICU admission, and delayed antibiotic treatment [10,13,27].

As supported by previous research, Legionnaires’ disease tends to occur more frequently during the summer months. This is likely due to the close association of Legionella with water, and the increased use of showers and air conditioning systems during this time. It is important to consider both environmental and social factors when addressing outbreaks of this disease. Regarding the results that identify non-drinking as a risk factor for mortality, it should be noted that our study only analyzed the status of alcohol consumption without considering the intensity or frequency of drinking. However, many studies have shown that low or moderate alcohol intake is not associated with mortality [28,29], with some studies even suggesting a protective effect of drinking against death [30,31]. In our study, we only distinguished between drinkers (current drinkers) and nondrinkers (former and never-drinkers) without quantifying the amount; hence, we cannot determine the association between the amount of alcohol consumption and mortality. Nevertheless, considering that the subjects of our study were mostly older adults and had underlying diseases, the number of high-risk drinkers is assumed to be low. It can be, thus, speculated that if the drinkers consumed alcohol in small amounts, their risk of death could be lower.

The lower risk of death among those who received antibiotics for more than 3 days could be due to patients who did not receive timely treatment after admission dying within a short period, while survivors continued to receive treatment beyond 3 days. The same results were observed when risk factor analysis was performed based on underlying diseases alone, both among patients and suspected cases. Further research is necessary to investigate the impact of alcohol consumption and duration of antibiotic use on mortality rates.

This study has the following limitations. Most of the epidemiological investigation reports did not include test results and diagnosis details. The investigation into the variables such as smoking and drinking history was based on patient statements, making objective verification impossible. Although additional epidemiological investigations were conducted on the environment after estimating the infection routes, it was not possible to analyze the descriptively entered details of environmental investigation. There were also difficulties in generalizing the risk factors of death due to the small number of deaths. Future studies with a broader scope or longer follow-up period will be able to generalize about mortality risk factors with greater confidence and accuracy.

Despite these limitations, this study is significant for shedding light on factors influencing the mortality rate of Legionellosis.

## 5. Conclusions

Nosocomial infections, a time lag of 1 to 3 days from admission to diagnosis, and the development of complications were significantly associated with the mortality rate of LD. Hospitals that accommodate patients with underlying diseases and ICUs must prioritize infection control measures to prevent outbreaks of Legionnaires’ disease. This includes promptly administering antibiotics, managing cooling towers, and managing water systems. As the population ages and the climate warms, the incidence and mortality of Legionnaires’ disease are likely to increase. Ongoing research, national health policy development, and application in clinical practice are necessary to eliminate risk factors of death.

## Figures and Tables

**Table 1:** Legionnaires’ cases definition.

	Cases Definition
**Confirmed** **cases**	Persons who have clinical symptoms characteristic of legionellosis and meet the laboratory testing criteria for a confirmed diagnosis. At least one of the following three: Isolation of Legionella bacteria from specimens such as bronchoalveolar lavage, bronchial aspirate, sputum, lung tissue, pleural fluid, and blood. Detection of specific antigens in urine samples. A ≥ fourfold increase in antibodies in convalescent serum compared to the acute phase.
**Suspected** **cases**	Persons who are suspected to have legionellosis based on clinical symptoms and epidemiological relevance, and presumed to be infected with Legionella based on the laboratory testing criteria for a suspected diagnosis. At least one of the following three: Detection of specific antigens in specimens using direct fluorescence antibody tests. A single antibody titer of 1:128 or higher in blood samples using indirect fluorescence antibody tests. Detection of specific genes in the specimen.

**Table 2:** Basic characteristics of the survivors and non-survivors among confirmed and suspected Legionnaires’ cases in Gyeonggi-do from 2016 to 2022.

Characteristics	Total			Confirmed Cases			Suspected Cases		
	Survivors	Nonsurvivors	*p*-Value	Survivors	Nonsurvivors	*p*-Value	Survivors	Nonsurvivors	*p*-Value
	*N* (%)	*N* (%)		*N* (%)	*N* (%)		*N* (%)	*N* (%)	
**Sex**									
Male	360 (89.33)	43 (10.67)	0.478	183 (89.71)	21 (10.29)	0.883	177 (88.94)	22 (11.06)	0.264
Female	128 (91.43)	12 (8.57)		57 (89.06)	7 (10.94)		71 (93.42)	5 (6.58)	
**Age**									
Mean (SD)	67.4	71.6	0.192	64.8	68.5	0.032	70	74.8	0.003
<50	37 (97.37)	1 (2.63)		22 (95.65)	1 (4.35)		15 (100.00)	0 (0.00)	
50–59	105 (93.75)	7 (6.25)		68 (95.77)	3 (4.23)		37 (90.24)	4 (9.76)	
60–69	112 (88.89)	14 (11.11)		54 (83.08)	11 (16.92)		58 (95.08)	3 (4.92)	
70–79	131 (86.75)	20 (13.25)		57 (87.69)	8 (12.31)		74 (86.05)	12 (13.95)	
80–	103 (88.79)	13 (11.21)		39 (88.64)	5 (11.36)		64 (88.89)	8 (11.11)	
**Route of infection**									
Nosocomial infection	59 (79.73)	15 (20.27)	0.001	30 (78.95)	8 (21.05)	0.026	29 (80.56)	7 (19.44)	0.070
Community infection	257 (93.45)	18 (6.55)		121 (93.80)	8 (6.20)		136 (93.15)	10 (6.85)	
Others	172 (88.66)	22 (11.34)		89 (88.12)	12 (11.88)		83 (89.25)	10 (10.75)	
**Current alcohol drinking**									
No	375 (88.03)	51 (11.97)	0.006	161 (86.56)	25 (13.44)	0.016	214 (89.17)	26 (10.83)	0.221
Yes	113 (96.58)	4 (3.42)		79 (96.34)	3 (3.66)		34 (97.14)	1 (2.86)	
**Current smoking status**									
No	343 (89.32)	41 (10.68)	0.510	145 (86.83)	22 (13.17)	0.061	198 (91.24)	19 (8.76)	0.252
Yes	145 (91.19)	14 (8.81)		95 (94.06)	6 (5.94)		50 (86.21)	8 (13.79)	
**Comorbidities**									
Diabetes	162 (90.50)	17 (9.50)	1	103 (90.35)	11 (9.65)	1	59 (90.77)	6 (9.23)	1
Cancer	114 (84.44)	21 (15.56)		46 (80.70)	11 (19.30)		68 (87.18)	10 (12.82)	
Renal failure	66 (84.62)	12 (15.38)		38 (90.48)	4 (9.52)		28 (77.78)	8 (22.22)	
Immunosuppressive medication	85 (89.47)	10 (10.53)		40 (88.89)	5 (11.11)		45 (90.00)	5 (10.00)	
Transplantation	22 (91.67)	2 (8.33)		16 (100.00)	0 (0.00)		6 (75.00)	2 (25.00)	
Others	366 (90.59)	38 (9.41)		167 (89.78)	19 (10.22)		199 (91.28)	19 (8.72)	
Any of ≥1 comorbidities	431 (89.23)	52 (10.77)		203 (88.26)	27 (11.74)		228 (90.12)	25 (9.88)	
**Symptoms**									
Fever	379 (91.55)	35 (8.45)	0.059	200 (90.50)	21 (9.50)	0.238	179 (92.75)	14 (7.25)	0.150
Cough	292 (90.68)	30 (9.32)		138 (90.79)	14 (9.21)		154 (90.59)	16 (9.41)	
Dyspnea	235 (84.84)	42 (15.16)		105 (84.00)	20 (16.00)		130 (85.53)	22 (14.47)	
Others	381 (90.07)	42 (9.93)		191 (89.25)	23 (10.75)		190 (90.91)	19 (9.09)	
Any of ≥1 symptoms	485 (89.98)	54 (10.02)		239 (89.51)	28 (10.49)		246 (90.44)	26 (9.56)	
**Admission to clinics**									
No	10 (100.00)	0 (0.00)	0.609	3 (100.00)	0 (0.00)	1	7 (100.00)	0 (0.00)	1
Yes	478 (89.68)	55 (10.32)		237 (89.43)	28 (10.57)		241 (89.93)	27 (10.07)	
**Admission to intensive care unit (*n* = 497)**									
No	320 (94.96)	17 (5.04)	<0.001	152 (94.41)	9 (5.59)	<0.001	168 (95.45)	8 (4.55)	<0.001
Yes	126 (78.75)	34 (21.25)		66 (79.52)	17 (20.48)		60 (77.92)	17 (22.08)	
**Prescription of antibiotics (*n* = 507)**									
No	12 (100.00)	0 (0.00)	0.620	4 (100.00)	0 (0.00)	1	8 (100.00)	0 (0.00)	1
Yes	444 (89.70)	51 (10.30)		219 (89.39)	26 (10.61)		225 (90.00)	25 (10.00)	
**Dual infection (*n* = 503)**									
No	321 (91.71)	29 (8.29)	0.022	172 (91.01)	17 (8.99)	0.062	149 (92.55)	12 (7.45)	0.118
Yes	130 (84.97)	23 (15.03)		46 (82.14)	10 (17.86)		84 (86.60)	13 (13.40)	
**Days from symptom onset to diagnosis**									
Mean (SD)	14.3	8.6		18.4	6.1		13.7	11.1	
Median (IQR)	7	6		6	4		8	6	
<5 days	130	22		83	15		47	7	
≥5 days	358	33		157	13		201	20	
**Days from diagnosis to admission among patients who were admitted to clinics (*N* = 533)**									
Mean (SD)	4.99	5.67		3.86	4.57		6.10	6.81	
Median (IQR)	3	3		2	1.5		4	4	
<3 days	203	23		135	17		68	6	
≥3 days	275	32		102	11		173	21	
**Duration of admission among patients who were admitted to clinics (*N* = 533)**									
Mean (SD)	11.06	8.80		9.46	9.17		12.63	8.40	
Median (IQR)	8	6		7	6		9	5	
<7 days	151	30		88	16		63	14	
≥7 days	327	25		149	12		178	13	
**Duration of antibiotics prescription among who were ever prescribed to antibiotics (*N* = 492)**									
Mean (SD)	11.32	7.70		9.03	8.12		13.54	7.28	
Median (IQR)	9	5		8	5		10	4	
<7 days	142	30		83	15		59	15	
≥7 days	300	20		134	10		166	10	

SD standard deviation; IQR interquartile range.

**Table 3:** Factors associated with survival among confirmed and suspected Legionnaires’ cases in Gyeonggi-do from 2016 to 2022.

Characteristics	Univariate Analysis			Multivariate Analysis		
	HR	95% CI	*p*-Value	HR	95% CI	*p*-Value
**Sex**						
Male	1			1		
Female	0.81	0.43–1.54	0.53	0.54	0.27–1.10	0.09
**Age**						
1 year increment	1.02	1.00–1.04	0.12	1.01	1.00–1.03	0.22
**Route of infection**						
Community infection	1			1		
Nosocomial infection	3.10	1.12–8.56	<0.001	5.23	1.72–15.94	0.004
Others	1.81	0.71–4.62	0.029	2.53	0.97–7.14	0.06
**Current alcohol drinking**						
No	1			1		
Yes	0.27	0.10–0.74	0.011	0.17	0.06–0.57	0.003
**Current smoking status**						
No	1			1		
Yes	0.93	0.51–1.72	0.93	1.65	0.80–3.37	0.17
**Comorbidities ^1^**						
No	1			1		
Yes	1.66	0.60–4.60	0.33	0.90	0.29–2.79	0.86
**Dual infection**						
No	1			1		
Yes	1.61	0.93–2.79	0.10	1.66	0.93–2.97	0.09
**Days from symptom onset to diagnosis**						
Per 1 day increment	1.00	0.97–1.02	0.57	1.00	0.97–1.02	0.61
**Days from diagnosis to admission**						
No admission	1			1		
1–3 days	11.03	4.02–30.21	<0.005	4.39	1.29–14.90	0.02
3 days or more	0.93	0.53–1.63	0.79	1.05	0.57–1.93	0.89
**Duration of antibiotic use**						
<3 days	1			1		
≥3 days	0.34	0.19–0.61	<0.001	0.17	0.08–0.39	<0.005
**Complications**						
No	1			1		
Yes	4.40	2.19–8.86	<0.005	5.46	2.64–11.28	<0.005
Undetermined	5.14	1.60–16.54	0.006	4.86	1.42–16.67	0.01

^1^ Comorbidities included diabetes, cancer, renal failure, diseases with immunosuppressive medication, transplantation, and others.

## Data Availability

The data used in this study are protected under the Personal Information Protection Act.
